# Interleukin-18 expression increases in response to neurovascular damage following soman-induced status epilepticus in rats

**DOI:** 10.1186/s12950-015-0089-9

**Published:** 2015-07-22

**Authors:** Erik A. Johnson, Michelle A. Guignet, Thuy L. Dao, Tracey A. Hamilton, Robert K. Kan

**Affiliations:** Research Division, Pharmacology Branch, U.S. Army Medical Research Institute of Chemical Defense (USAMRICD), Aberdeen Proving Ground, MD 21010-5400 USA; Research Support Division, Comparative Pathology Branch, U.S. Army Medical Research Institute of Chemical Defense (USAMRICD), Aberdeen Proving Ground, MD 21010-5400 USA

**Keywords:** Interleukin 18, Status epilepticus, Soman (GD), Macrophage, T-cell, Neutrophil, Piriform cortex, Hippocampus, Thalamus

## Abstract

**Background:**

Status epilepticus (SE) can cause neuronal cell death and impaired behavioral function. Acute exposure to potent acetylcholinesterase inhibitors such as soman (GD) can cause prolonged SE activity, micro-hemorrhage and cell death in the hippocampus, thalamus and piriform cortex. Neuroinflammation is a prominent feature of brain injury with upregulation of multiple pro-inflammatory cytokines including those of the IL-1 family. The highly pleiotropic pro-inflammatory cytokine interleukin-18 (IL-18) belongs to the IL-1 family of cytokines and can propagate neuroinflammation by promoting immune cell infiltration, leukocyte and lymphocyte activation, and angiogenesis and helps facilitate the transition from the innate to the adaptive immune response. The purpose of this study is to characterize the regional and temporal expression of IL −18 and related factors in the brain following SE in a rat GD seizure model followed by localization of IL-18 to specific cell types.

**Methods:**

The protein levels of IL-18, vascular endothelial growth factor and interferon gamma was quantified in the lysates of injured brain regions up to 72 h following GD-induced SE onset using bead multiplex immunoassays. IL-18 was localized to various cell types using immunohistochemistry and transmission electron microscopy. In addition, macrophage appearance scoring and T-cell quantification was determined using immunohistochemistry. Micro-hemorrhages were identified using hematoxylin and eosin staining of brain sections.

**Results:**

Significant increases in IL-18 occurred in the piriform cortex, hippocampus and thalamus following SE. IL-18 was primarily expressed by endothelial cells and astrocytes associated with the damaged neurovascular unit. The increase in IL-18 was not related to macrophage accumulation, neutrophil infiltration or T-cell appearance in the injured tissue.

**Conclusions:**

These data show that IL-18 is significantly upregulated following GD-induced SE and localized primarily to endothelial cells in damaged brain vasculature. IL-18 upregulation occurred following leukocyte/lymphocyte infiltration and in the absence of other IL-18-related cytokines, suggesting another function, potentially for angiogenesis related to GD-induced micro-hemorrhage formation. Further studies at more chronic time points may help to elucidate this function.

## Background

Exposure to chemical warfare nerve agents (CWNA), such as soman (pinacolyl methylphosphonofluoridate, GD), causes a myriad of negative physiological events related to excess acetylcholine accumulation to include intense tonic-clonic convulsions, acute morbid hypertension, and potentially death [1–3]. In the brain, nerve agent exposure causes status epilepticus (SE) [4] leading to excitotoxic brain injury, neuroinflammatory gliosis, neutrophil infiltration, expression of pro-inflammatory factors and widespread neuronal cell loss observed in the piriform cortex, hippocampus, amygdala and thalamus [5–8]. In addition, acute hypertension can damage capillaries, further exacerbating brain injury. In response, innate inflammatory processes occur to include upregulation of interleukin-1 (IL-1) family cytokines (IL-1α and β), leukocyte chemotaxis factors (CXCL1 and MIP-1α) and leukocyte recruitment in injured brain regions [8, 9]. IL-18 is a member of the IL-1 family of cytokines that is involved in both innate and adaptive immune responses and helps transition a non-specific inflammation reaction to a directed lymphocyte response [10]. Because of this pleiotropic nature, IL-18 has been implicated in several injury-related processes including immune cell activation, propagation of the adaptive immune response and vascular repair.

Unlike most pro-inflammatory cytokines, IL-18 appears to be constitutively expressed in the brain [11–13]. However, IL-18 significantly increases expression in damaged brain cells such as neurons [14] and endothelial cells [15], and injury response cells such as astrocytes [14, 16], microglia [16, 17], macrophages [18] and infiltrating monocytes [19] in response to various brain insults including seizure [14, 20, 21]. IL-18 is particularly involved in guiding and activating circulating immune cells to injured tissues. IL-18 can induce factors involved in neutrophil chemotaxis such as CXCL1 and leukotriene B4 [22, 23] and can upregulate various cellular adhesion molecules on monocytes [22, 24], T-cells [25] and endothelial cells [15, 22, 26] to facilitate rolling and vascular infiltration. IL-18 expression promotes immune cell activation by inducing the expression of multiple pro-inflammatory cytokines, chemokines and proliferative factors in activated macrophages [27–29], microglia [17], CD4(+) T-cells [28], B cells [30], natural killer (NK) cells [31, 32], CD8+ lymphocytes [17] and neutrophils [23]. IL-18 plays an important role in transitioning innate inflammation to the adaptive immune response by providing an early signal for development of Th1 lymphocyte responses [33] and by helping regulate functionally distinct subsets of T-helper cells required for cell-mediated immune responses [34]. In conjunction with IL-12 [32], IL-18 expression enhances the adaptive immune response particularly through increased expression and release of interferon gamma (IFNγ) by T- and NK cells [31], which in turn upregulates and promotes Fas-mediated apoptosis [35]. Lastly, IL-18 also affects angiogenesis through induction of vascular endothelial growth factor (VEGF) [36] and by stimulating endothelial cell proliferation and chemotaxis to repair damaged blood vessels [37]. In conjunction with IL-12, IL-18 can also upregulate angiogenic and angiostatic chemokines, which also contribute to the wound healing process [38].

While early innate mechanisms of inflammation have been investigated following seizurogenic nerve agent exposure, IL-18 is unique as an immune transition cytokine to a directed adaptive response. Lymphocyte infiltration following ‘sterile’ neuroinflammation (i.e., inflammation not caused by infection) has been reported following various CNS injuries including acute ischemic damage [39], though the exact role these cells play in models of injury is not well understood. However, a lack of T-cell response (both CD4+ and CD8+) can attenuate brain damage in various CNS injury models [40]. Because of the ‘sterile’ nature of GD-induced seizure injury, it is not clear whether IL-18 plays a role in the progression of the inflammatory response to a more chronic response following nerve agent exposure or whether it has a more specific role in acute response to injury (e.g., leukocyte infiltration). Therefore, the purpose of this study was to investigate IL-18 upregulation and related factors (i.e., VEGF, IFNγ and T-cell related interleukins) following GD exposure, using multiplex bead immunoassay and immunohistochemistry. Further studies were conducted to localize IL-18 expression to areas of vascular injury and to investigate the innate to adaptive immune response transition using macrophage and T-cell infiltration into injured brain areas. These data are the first to show IL-18 upregulation and cellular origin along with expression of other innate-to-adaptive immune response factors following GD-induced SE.

## Methods

### Animals and GD Seizure Model

Adult male Sprague–Dawley rats (Charles River Laboratories, Wilmington, MA; CRL: CD[SD]-BR, 250–350 g) were treated with HI-6 dichloride (Walter Reed Army Institute of Research, Silver Spring, MD; 125 mg/kg, i.p.) 30 min prior to GD administration and with atropine methyl nitrate (AMN, Sigma-Aldrich, St. Louis, MO; 2.0 mg/kg, i.m.) 1 min after GD administration. Vehicle control animals received HI-6, AMN and saline, while naïve animals received no injections. GD (GD-U-2323-CTF-N, purity 98.8 wt%) was diluted in saline at the USAMRICD. GD (180 μg/kg) was administered subcutaneously in the scruff of the neck, and the rat was observed for convulsive activity. This dose of GD produces a 100 % generalized convulsive seizure rate within minutes that is maintained up to 24 h [41, 42]. The experimental protocol was approved by the Animal Care and Use Committee at the United States Army Medical Research Institute of Chemical Defense and all procedures were conducted in accordance with the principles stated in the Guide for the Care and Use of Laboratory Animals (National Research Council, 2011), and the Animal Welfare Act of 1966 (P.L. 89–544), as amended. The animal care program at this institute is fully accredited by the Association for Assessment and Accreditation of Laboratory Animal Care, International.

### Multiplex bead array immunoassay

Experimental and vehicle control animals were euthanized with a sodium pentobarbital solution (65–100 mg/kg, i.p.) at 0.5, 1, 3, 6, 12, 24, 48 or 72 h after onset of convulsions; naïve animals were euthanized at 24 h only. Following euthanasia, the brains were removed, dissected and processed into lysate as previously described [9]. Briefly, the piriform cortex, hippocampus and thalamus were excised, rinsed with cold PBS and snap frozen in liquid nitrogen. The tissues were weighed and homogenized in ice-cold triple detergent lysis buffer containing a Complete™ protease inhibitor cocktail (Roche Biochemicals, Indianapolis, IN) at a ratio of 1 ml buffer to 50 mg tissue. Samples were allowed to stand at 4 ° C for at least 30 min before centrifugation at 8000 G for 5 min and removal of the lysate for assaying. IL-18, VEGF and IFNγ concentrations were quantified using a rat cytokine multiplex bead immunoassay kit (EMD Millipore, St. Charles, MO). Concentrations of IL-4, IL-10, IL-12 and IL-17 were run in parallel and previously reported [8]. The bead immunoassay procedure used 25 μl of lysate (94 ± 8 μg protein) per well and was conducted according to the manufacturer’s instructions. The plate was read on a Luminex™ 100 instrument (Bio-Rad Laboratories, Hercules, CA) and analyzed with either BioRad or STaRStation software (Applied Cytometry, Sacramento, CA). Experimental and naïve group numbers were ≥5 for all brain regions and time points except for the experimental 6 h (*n* = 4) and 12 h (*n* = 3) thalamus. Time-matched vehicle controls (*n* = 3 per time point) were analyzed individually and pooled into a single vehicle control comparison group when no significant difference was found between these samples over time by analyte or brain region (data not shown).

### Immunohistochemistry (IHC)

Separate from the animals used in the multiplex bead array immunoassay, experimental, vehicle control and naïve animals were deeply anesthetized and perfused with cold isotonic saline followed by 4 % paraformaldehyde via cardiac puncture. Brains were moved to 4 % formaldehyde for 6–8 h, then allowed to sit in 30 % sucrose/PBS at 4 ° C for another 12–16 h. Tissues were then blocked, cut on a Leica CM3050 S cryostat (Thermo Shandon, Inc.; Pittsburgh, PA) at 40 μm and stored in cryobuffer (30 % each of glycerol, ethylene glycol and water, 10 % 2 × phosphate buffer) until use. Free float fluorescent IHC labeling was conducted as previously described [43]. Experimental and vehicle control samples had an *n* = 4 for each cytokine/cell type combination. The antibodies used were as follows: rabbit anti-IL-18 (1:250; sc6179, Santa Cruz Bio, Santa Cruz, CA), mouse anti-NeuN (1:1000; MAB377, Chemicon, Temecula, CA), mouse anti–glial fibrillary acidic protein (GFAP)(1:1000; MS-280-P, NeoMarkers, Fremont, CA), mouse anti-cd11b (1:1000; CBL1512, Chemicon), mouse anti-rat endothelial cell antigen (RECA)(1:1000; ab9774, Abcam), mouse anti-von Willebrand factor (vWf)(1:100; MAB3442, Chemicon), rabbit anti-smooth muscle alpha actin (1:250, Abcam), and mouse anti-NG2 chondroitin sulfate (1:500; MAB5384, Millipore). For anti-NeuN, anti-GFAP, anti-cd11b, anti-RECA, anti-von Willebrand factor, anti-smooth muscle alpha actin and anti-NG2 chondroitin sulfate, a species-appropriate Alexafluor™ 594 fluorescent-tagged secondary (1:1000, Molecular Probes; Eugene, OR) was used for visualization. For anti-IL-18, a biotin-labeled secondary (1:1000, Vector Laboratories; Burlingame, CA) followed by an Alexafluor™ 488 fluorescent-tagged tertiary antibody (1:1000, Molecular Probes) was used for signal amplification. Tissue sections labeled with only secondary and tertiary antibodies were used as controls. All sections used were located between interaural 6.20 mm, bregma −2.80 mm and interaural 5.20 mm, bregma −3.80 mm [44]. For experiments requiring cell counting, investigators were blinded from the conditions. Sections were viewed and digitally captured with an Olympus BX51 microscope equipped with an Olympus DP-70 high-resolution color CCD digital camera. An Olympus BX61 equipped with a DSU spinning disk confocal system and DP-70 CCD camera was used to confirm same cell co-localization. Images of 40 μm tissues were acquired using a z step interval of 1 μm and analyzed using Slidebook™ software. Publication images were compiled using Adobe Photoshop CS4 digital image software. To more easily view the nuclei in 10x photomicrographs, the blue channel had the unsharpen mask filter applied with the following parameters: Amount = 500 %, Radius = 2.6 pixels, Threshold = 13 levels. For all photomicrographs, color levels were evened using the ‘levels’ tool. Input levels (0–255) were normalized in the RGB channel as follows: highlight input levels were set at the peak of the image histogram (10x and 40x), midtone levels were set at 0.8 (10x and 40x) and shadow levels were set at 75 (10x) or at the edge of the histogram closest to 255 or at 180, whichever was greater (40x).

### Transmission electron microscopy

Free floating brain sections approximately 40 μm thick were labeled with rabbit anti-IL-18 and biotinylated anti-rabbit IgG secondary as described above and incubated in avidin-biotin-peroxidase complex solution (Life Technologies; Grand Island, NY) at room temperature for 30 min and finally with SIGMAFast™ 3,3′-diaminobenzidine (DAB; Sigma-Aldrich, St. Louis, MO) solution for desired stain intensity. Regions of interest were excised from the tissue, buffer-washed, post-fixed in buffered 1 % osmium tetroxide, dehydrated in graded ethanol and embedded in PolyBed® epoxy resin (Polysciences Inc., Warrington, PA). Ultrathin tissue sections approximately 90 nm thick were mounted on copper mesh grids. Imaging was performed using a JEOL JEM-1230 transmission electron microscope (JEOL USA Inc.; Peabody, MA).

### Histology for micro-hemorrhage

Tissue samples were fixed in 10 % formalin, dehydrated in a series of alcohol washes, cleared in a xylene substitute (Micro-Clear, Micron Environmental Industries, Alexandria, VA) and embedded in paraffin. Sections were cut at 5 μm, stained with hematoxylin and eosin (H&E) using the Leica ST5020 Multistainer - CV5030 Coverslipper integrated workstation (Leica Biosystems Inc., Buffalo Grove, IL) and digitally captured using the Nanozoomer 2.0RS slide scanner (Olympus). Micro-hemorrhage was defined as ≥5 red blood cells found in the tissue around a blood vessel.

### Macrophage appearance scoring

Sections from vehicle control and experimental time points at 0.5, 1, 3, 6, 12, 24 and 48 h after onset of convulsions labeled with anti-cd11b (described above) were used for this analysis. Three representative 10x micrographs (2.44 × 1.06 μm^2^) were taken from ≥ 3 animals for each brain region/animal; the median was used for data analysis. Macrophages were identified as a cell having ameboid morphology, showing a predominant loss of processes and being positive for cd11b. A macrophage scoring system was developed based on number of macrophages present per micrograph. Criteria for scoring were as follows: Score (0), No ameboid cells present; Score (1), 1–10 ameboid cells present throughout 10x photomicrograph; Score (2), 11–29 ameboid cells present throughout 10x photomicrograph; and Score (3), >30 ameboid cells present throughout 10x photomicrograph. Origin of macrophages, either central or peripheral, was not determined.

### T-cell quantification

Sections from vehicle control and experimental time points 6, 12, 24, 48 and 72 h after onset of convulsions were labeled with mouse anti-rat pan T-cell marker (1:100; sc-52711, Santa Cruz) and visualized with DAB. Images were visualized and captured as described above. Bilateral representative 10x micrographs (2.44 × 1.06 μm^2^) were taken for each brain region/animal with each time point having an n =3. T-cells were quantified using Visiopharm software by counting positively labeled cells per region/mm^2^. Counts were divided by cells bound to the luminal side of the vasculature and those that had infiltrated into the tissue.

### Statistical analysis

Temporal changes in immunoassay data and T-cell quantification were evaluated by ANOVA with a post-hoc Bonferroni’s analysis. Macrophage appearance scores were evaluated using a Kruskal-Wallis test followed by a post-hoc Dunn’s Multiple Comparison test. Pearson’s correlation coefficients were calculated for macrophage appearance scoring, and T-cell quantification data compared to IL-18 concentration data. Values are expressed as mean ± SEM. For immunoassay data, points calculated below the minimum detectible concentrations (MinDC) for the assays were conservatively set at −0.01 pg/ml of the MinDC for statistical analyses. Differences were considered significant at the level of p ≤ 0.05. All statistics were analyzed using GraphPad Prism v5.04 for Windows.

## Results

### IL-18 is significantly upregulated in the brain following GD-induced seizure

**Fig. 1:**
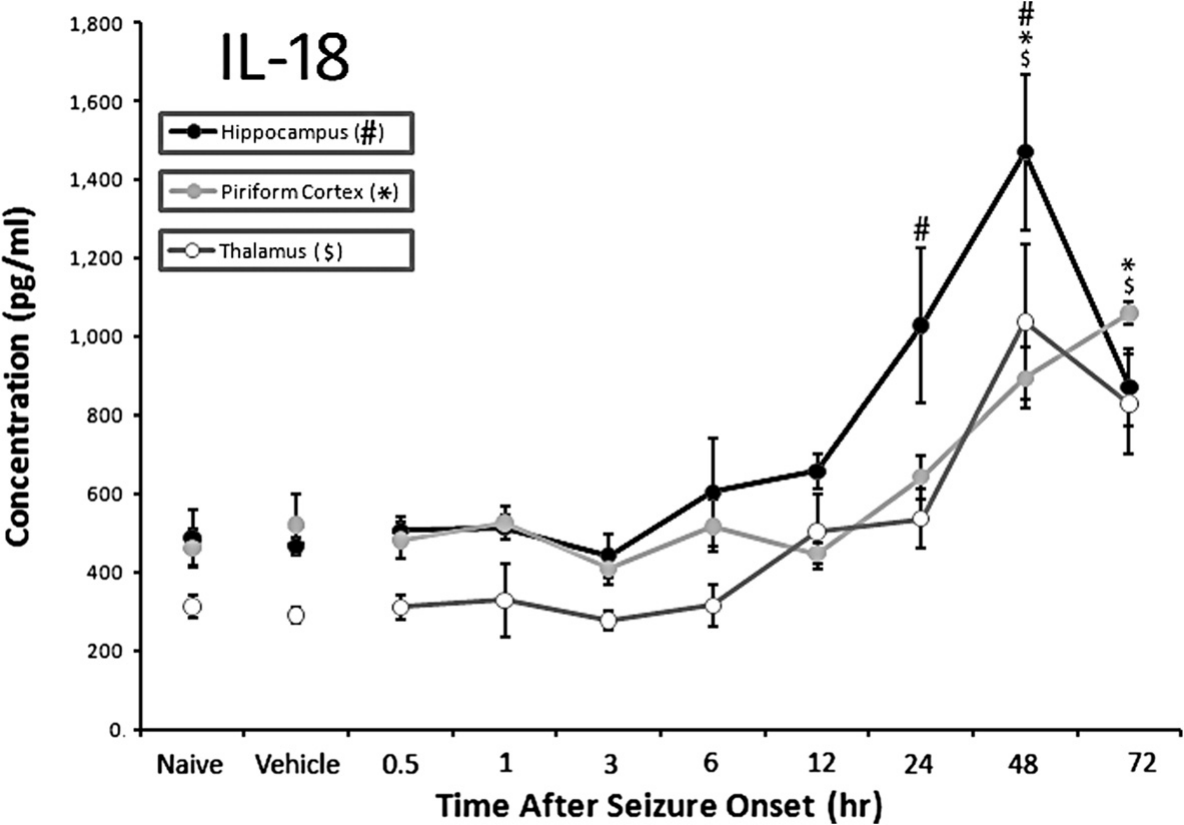
IL-18 is expressed in the rat brain following GD-induced SE. Concentrations of IL-18 peak at 48 h in the hippocampus (solid black line) and thalamus (open gray line), while the highest concentration in the piriform cortex (solid gray line) is at 72 h. Data are given as pg/ml of tissue lysate and reported as mean ± SEM. Statistical comparisons were to vehicle controls within each brain region using ANOVA with a post-hoc Bonferroni’s analysis. (# p < 0.05 hippocampus, * p < 0.05 piriform cortex, $ p < 0.05 thalamus)

Temporal and regional changes in IL-18, IFNγ and VEGF protein concentrations were determined using a bead-based multiplex immunoassay on tissue lysates from the piriform cortex, hippocampus and thalamus. IL-4, IL-10, IL-12 and IL-17 protein concentrations were investigated in previous experiments and did not significantly change [8]. Additionally, no significant changes were observed for IFNγ or VEGF in this experiment (data not shown). Significant changes in IL-18 concentrations were observed in the piriform cortex, hippocampus and thalamus up to 72 h following GD-induced seizure compared to vehicle controls (Fig. 1). The highest concentrations were in the hippocampus, where concentrations significantly increased by 24 h (1030 ± 197 pg/ml) and peaked by 48 h (1471 ± 197 pg/ml vs. 488 ± 30 pg/ml in vehicle controls) following GD-induced SE. In the piriform cortex, IL-18 levels were significantly elevated by 48 h (895 ± 78 pg/ml) and continued to rise through 72 h (1060 ± 28 pg/ml) compared to vehicle controls (521 ± 80 pg/ml). In the thalamus, IL-18 concentration peaked at 48 h (1039 ± 199 pg/ml) and was still significantly greater at 72 h (829 ± 128 pg/ml) compared to vehicle controls (290 ± 19 pg/ml).

### IL-18 is expressed primarily by endothelial cells in injured brain regions

**Fig. 2:**
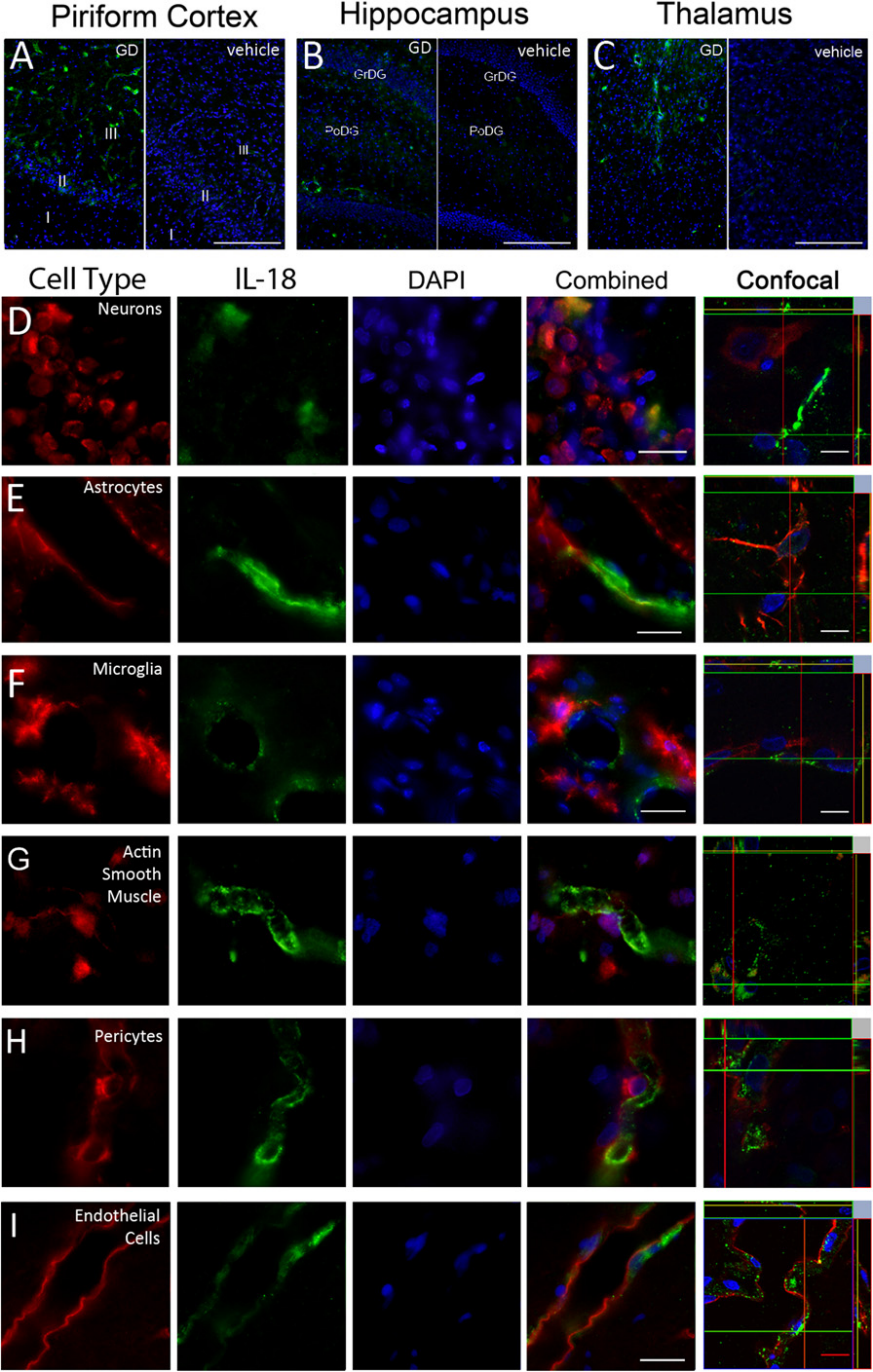
IL-18 expression is associated with vascular structures in injured brain regions. Tissue sections at 48 h were labeled with anti-IL-18 antibody along with cell type-specific antibodies. IL-18 immunolabeling (**a**-**i**, green) is present in the piriform cortex, hippocampus and thalamus following GD-induced SE (**a**, **b & c**; left) but absent in vehicle controls (**a**, **b & c**; right). IL-18 labeling was associated with the neurovascular unit, though labeling was predominantly co-incident with these immunolabels. Neurons (**d**, red) and vasculature-associated astrocytes (e, red) had rare and non-contiguous co-localization. Labeling was absent in microglia (**f**, red) and smooth muscle (**g**, red). Consistent co-incident labeling of IL-18 was present near pericytes (**h**, red) and endothelial cells (**i**, red). DAPI (**a**-**h**, blue) was used to label the nuclei of cells. (Scale bar: 250 μm [**a**-**c**], 50 μm for fluorescent and 5 μm [**d**-**i**] for confocal microscopy; *n* = 3 for 48-h, *n* = 4 for vehicle controls)

Forty-eight hours following GD-induced SE, IL-18 immuno-labeling was present in the piriform cortex (Fig. 2a, left), hippocampus (Fig. 2b, left) and thalamus (Fig. 2c, left). Specific labeling was largely absent in vehicle controls (Fig. 2a-c, right), though some sporadic labeling was observed in the piriform cortex. No specific labeling was observed with secondary-only or secondary/tertiary-only controls (data not shown).

**Fig. 3:**
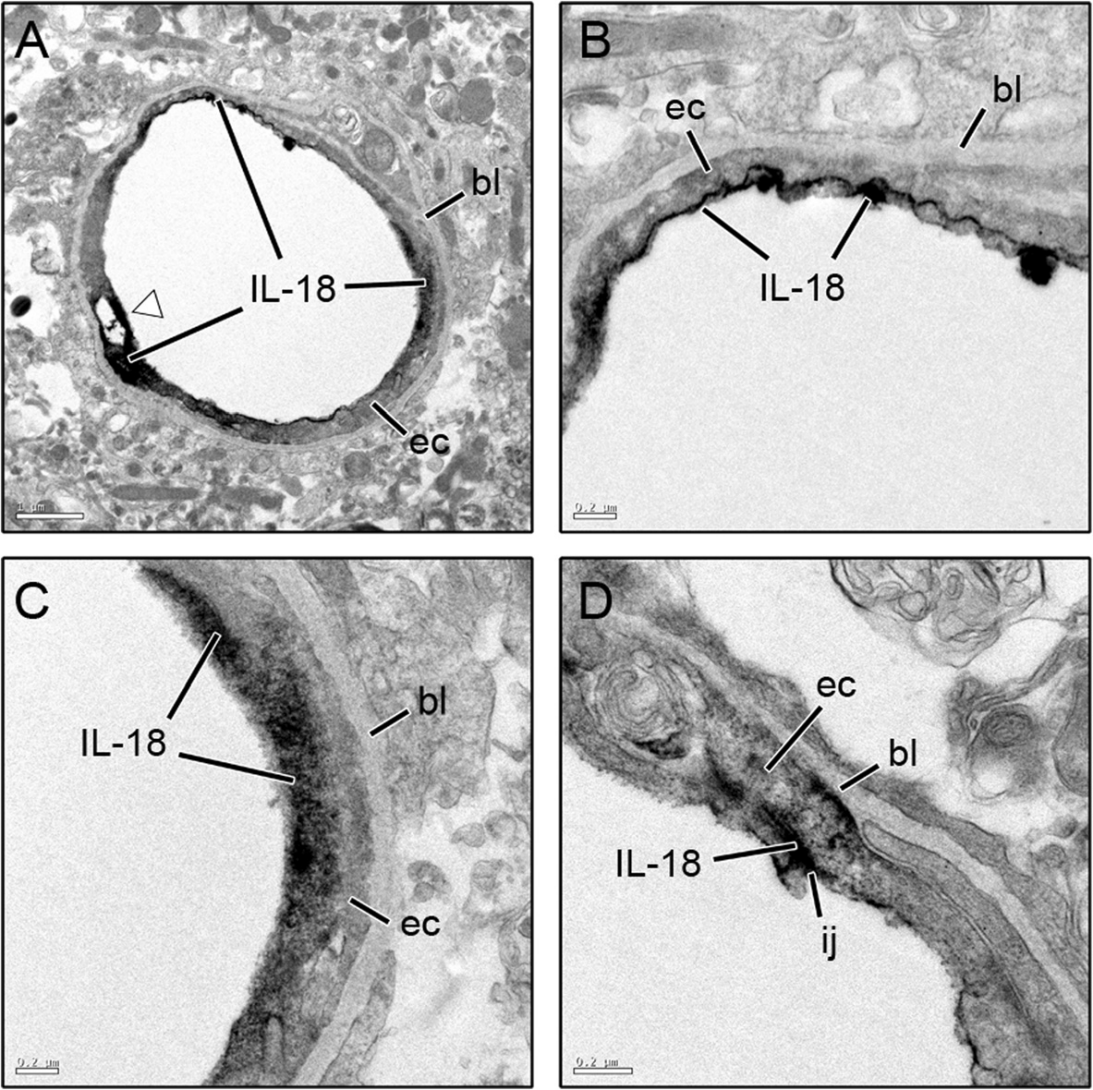
IL-18 is expressed primarily by endothelial cells. Transmission electron microscopy images of damaged piriform cortex shows IL-18 expression associated with endothelial cells (**a**-**d**). Expression was observed around damaged endothelial connections (a, open arrow), on the luminal surface (**b**), in the cell body (**c**) and at intracellular junctions of endothelial cells (**d**). (ec = endothelial cell, bl = basal lamina, ij = intracellular junction. Scale bar: 1 μm [a], 0.2 μm [b-d]; n = 3)

While some IL-18 labeling was observed in NeuN-positive neuronal populations in layer II of the piriform cortex (Fig. 2d), pyramidal neurons in the CA3 hippocampus, pyramidal and extrapyramidal neurons of the dentate gyrus and in neurons of the dorsolateral thalamus, the majority of IL-18 labeling was found in and around cells of the neurovascular unit. However, strict co-localization with cell type markers was sporadic and most often co-incident (i.e., the labels appeared in close proximity, typically adjacent). Few instances of co-localization were observed between IL-18 and GFAP-positive astrocytes (Fig. 2e), while no co-localization was found with cd11b-positive microglia (Fig. 2f) or smooth muscle labeled with actin (Fig. 2g). IL-18 appeared to be expressed by NG2 chondroitin sulfate-positive pericytes (Fig. 2h) and/or RECA-positive endothelial cells (Fig. 2i) because of the vascular morphology displayed by IL-18 and strong co-incident labeling with RECA, an antigen found only on the luminal side of endothelial cells. To confirm cell type expression of IL-18, transmission electron microscopy was used. IL-18 was only found within cells identified phenotypically as endothelial cells (Fig. 3a), typically around regions of vascular injury. IL-18 was primarily expressed in a defined layer towards the apical region of the endothelial cell (Fig. 3b) but could also be identified as diffuse labeling within the cell (Fig. 3c) and between the intracellular junctions (Fig. 3d).

### IL-18 is associated with GD-induced vascular damage

**Fig. 4:**
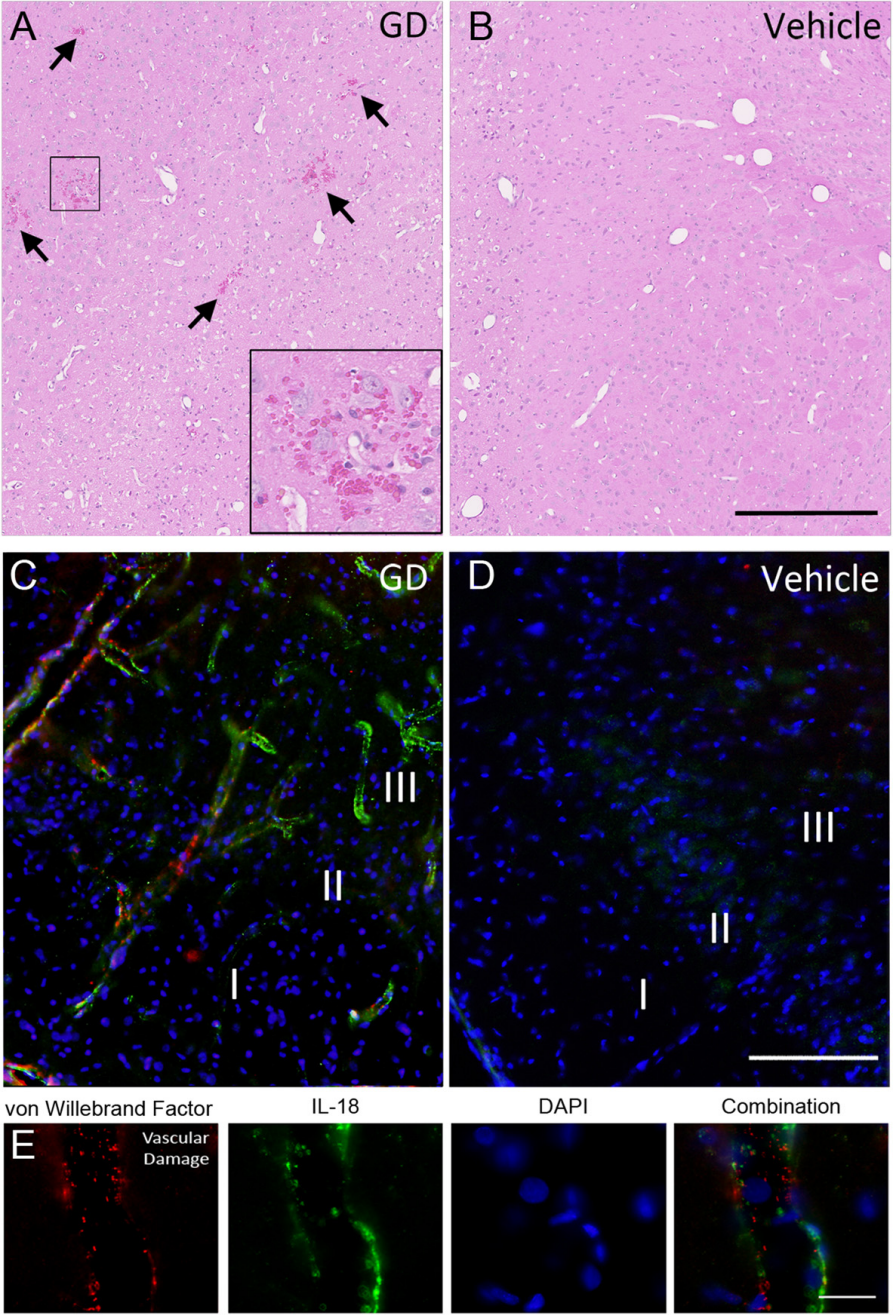
IL-18 expression is associated with vascular damage. GD exposure causes vascular damage and capillary micro-hemorrhages primarily in the thalamus (**a**), though it is also present in other brain regions. Multiple micro-hemorrhages were identified by areas of red blood cell infiltration in the tissue around capillaries (black arrows and inset). Vascular injury is absent in vehicle controls (**b**). Immunolabeling for the more sensitive endothelial cell injury protein, von Willebrand factor (vWf, red), showed extensive damage in the piriform cortex (**c**) and other brain regions after GD exposure along with IL-18 (green), but both were absent in vehicle controls (**d**). IL-18 and vWf labeling were often co-incident to the same vascular structures, though co-localization was rare (**e**), consistent with IL-18 expression in endothelial cells. (Scale bar: 250 μm [a-d], 50 μm for fluorescent [e]; *n* = 3 for 48-h, *n* = 4 for vehicle controls)

Following GD exposure, numerous capillary hemorrhages were observed by H&E staining in all damaged brain regions, though most prominently in the thalamic nuclei, starting at 3 h and persisting up to 72 h after seizure onset (Fig. 4a). No micro-hemorrhages were observed in vehicle controls (Fig. 4b). Damage was also visualized with an antibody to von Willebrand factor (vWf), a protein that binds to exposed collagen in endothelial cells in regions of vascular damage and is a more sensitive marker of vascular injury. IL-18 and vWf labeling were present in exposed piriform cortex (Fig. 4c) and absent in control brains (Fig. 4d). Fewer instances of vWf positive labeling were observed in the hippocampus and thalamus, consistent with H&E, though prominent IL-18 positive labeling was found in both of these regions. IL-18 and vWf labeling were often co-incident and co-localized to the same continuous vascular structure (Fig. 4e), though each protein could also readily be found localized to a vascular structure without the other present.

### IL-18 expression positively correlates with macrophage accumulation but not T-cell infiltration

**Fig. 5:**
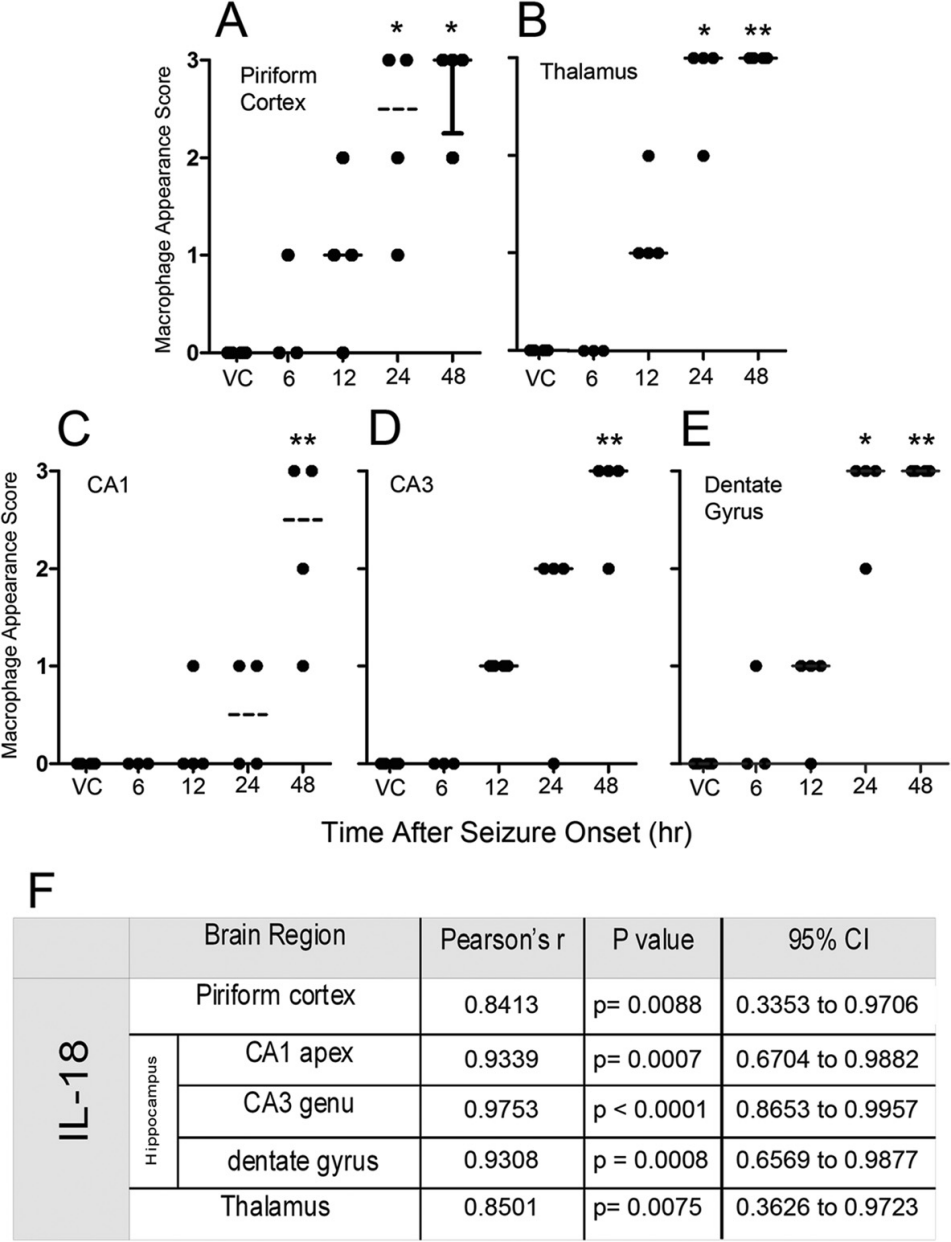
Macrophage appearance precedes significant IL-18 expression in damaged brain regions. Macrophages significantly appear at 24 h following seizure onset in the piriform cortex (**a**), thalamus (**b**), and dentate gyrus (**e**). Macrophages in the CA1 (**c**) and CA3 (**d**) significantly increase by 48 h. Pearson’s analysis shows a positive correlation between IL-18 expression and macrophage appearance in the CA1, CA3 and dentate gyrus regions of the hippocampus, thalamus and piriform cortex (**f**). Data are given as a macrophage appearance score and reported as median ± Q1 and Q3 and were analyzed using a Kruskal-Wallis test with a post-hoc Dunn’s Multiple Comparison test. (*n* = 3 or 4 for each time point/ brain region; * p < 0.05, ** p < 0.01)

IL-18 plays a role in monocyte infiltration and macrophage activation [22, 24, 27–29]. Therefore, the appearance of macrophages was scored in each brain region and correlated to the expression of IL-18 (Fig. 5). Significant accumulation of macrophages begins at 24 h following seizure onset in the piriform cortex (Fig. 5a), thalamus (Fig. 5b), and dentate gyrus (Fig. 5E). Significant macrophage accumulation begins in the CA1 (Fig. 5c) and CA3 (Fig. 5d) regions of the hippocampus at 48 h. No significant accumulation of macrophages was observed at earlier time points (data not shown). Significant Pearson’s correlation coefficients indicate that IL-18 expression and macrophage appearance were highly correlated in all brain regions investigated (Fig. 5f). However, significant macrophage accumulation precedes significant IL-18 expression in the three most injured regions (i.e., piriform cortex, thalamus and dentate gyrus).

**Fig. 6:**
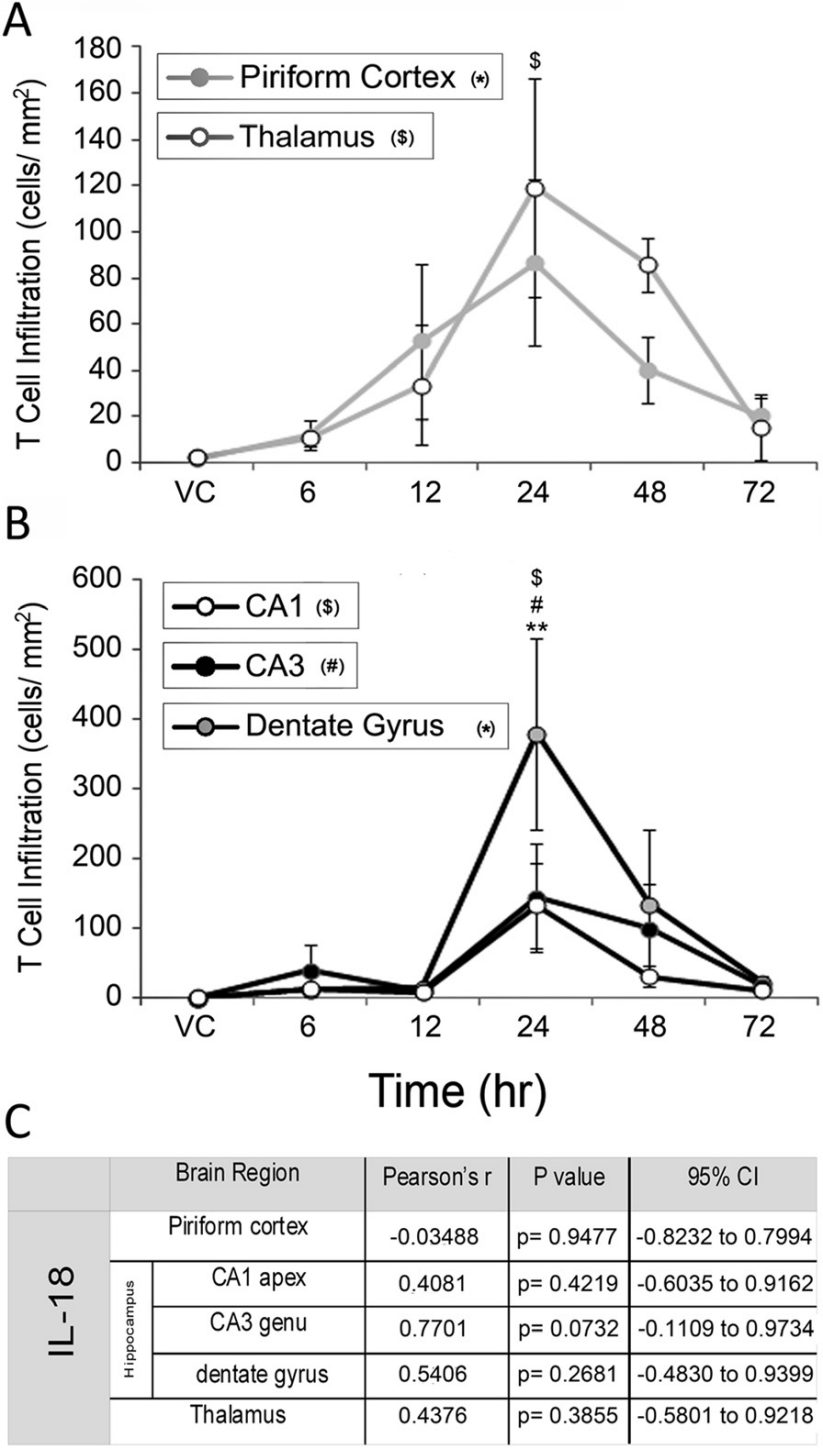
T-cell infiltration precedes significant IL-18 expression in damaged brain regions. Significant T-cell infiltration was observed at 24 h following GD-induced seizure activity in thalamus (**a**, open gray line), the CA1 (**b**, open black line), CA3 (**b**, solid black line) and dentate gyrus (**b**, black/gray line) though not in the piriform cortex (solid gray line, **a**). Increased T-cell infiltration preceded significant IL-18 expression, and no positive correlation was found between the two in any brain region (**c**). Data are given as cells/mm^2^ of tissue and reported as mean ± SEM and analyzed using a Kruskal-Wallis test with a post-hoc Dunn’s Multiple Comparison test. (*n* = 3 for each time point/ brain region; A: $ p < 0.05 thalamus, B: $ p < 0.05 CA1, # p < 0.05 CA3, ** p < 0.01 dentate gyrus)

T-cell infiltration into injured tissues was quantified in each brain region because of the ability of IL-18 to increase T-cell adhesion to endothelial cells associated with injury [25] (Fig. 6). A significant accumulation of T-cells occurred in the thalamus (Fig. 6a, open gray line), CA1 (Fig. 6b, open black line), CA3 (Fig. 6b, black line) and dentate gyrus (black/gray line) at 24 h. Though increases in T-cells were also seen in the piriform cortex (Fig. 6a, gray line), these were not significant. T-cells bound to the luminal side of the vasculature were also quantified, though no significant changes were observed (data not shown). IL-18 expression and T-cell infiltration are not significantly correlated in any brain region (Fig. 6c).

## Discussion

IL-18 is a unique pro-inflammatory cytokine that has been implicated in multiple facets of the neuroinflammatory response to include innate-to-adaptive immune response transitions, angiogenesis and leukocyte/lymphocyte infiltration. The function of IL-18 is still not well understood and is likely injury-specific; IL-18 expression appears to exacerbate pathology in some CNS injury models [45, 46] and reduce pathology in others [14, 21, 47, 48]. This study shows that IL-18, like other IL-1 family members [8], is expressed in response to GD-induced pathogenic seizure activity and vascular injury. IL-18 expression was prominent following GD-induced brain injury in the three brain regions investigated and peaked later than other IL-1 family members in this model [8]. The timing of expression is consistent with other studies including models of seizure induced by kainic acid (KA) [20], hypoxia-ischemia [45], traumatic brain injury [46] and neurovirulent influenza A virus infection [49]. However, in this study, IL-18 protein primarily localized to endothelial cells, perhaps as a result of pervasive neurovascular injury caused by GD exposure, whereas microglia and macrophages were the main producers of IL-18 in other CNS injury models [20, 45].

Previous studies have indirectly suggested a vascular injury component to CWNA exposure likely caused by hypertension and hypoxia [2, 50–52] and resolved by delayed angiogenesis via VEGF upregulation [53]. This study has shown that seizurogenic exposure to GD results in brain micro-hemorrhages and von Willebrand factor expression after GD exposure. IL-18 expression in response to these injuries can facilitate vascular repair by upregulating VEGF, stimulating new endothelial cell proliferation and chemotaxis to injured vessels [22, 36, 37]. Injury to the vasculature appears to stimulate expression of IL-18 in endothelial cells in this model as part of the angiogenic process after GD exposure [53]. VEGF, however, was not detected in the first 3 days after GD-induced injury in this model contrary to previous studies in mice [53]. However, it cannot be discounted that VEGF may appear just beyond the time course studied to increase mature blood vessel formation months after inury [53]. While outside the scope of this study, later time points may elucidate more of this potential function.

Activated microglia can migrate to injured and dying neurons following GD-induced seizure [9], though this study found little evidence of microglial or macrophage expression of IL-18 even as significant numbers of macrophages began to appear in the injured tissue. Because resident macrophages (i.e., from microglial lineage) are the first to respond and migrate to areas of injury before IL-18 expression, it is unlikely that this cytokine is required for chemotaxis during acute injury. However, IL-18 may be promoting monocyte infiltration and/or macrophage activation at the site of injury at later time points [45, 54]. Additionally, neurons express IL-18 receptor in response to injury [45], including seizure activity [14], and can increase IFNγ expression as a result of IL-18 signaling [49]. However, it is unlikely that IL-18 expression affects neurons shortly after injury as no increases in IFNγ were observed in the injured brain regions. While it is possible that IL-18 expression may originate from macrophages as the neuroinflammatory cascade progresses and this may affect IFNγ expression in neurons, the expression observed in this model appears to be in response to primary vascular injury.

Immune cell infiltration allows circulating phagocytic cells such as neutrophils and monocytes to enter the brain and begin to remove debris as one of the first steps of the healing process. Lymphocytes, particularly CD4+ and CD8+ T-cells, can also enter the injured brain and can influence injury resolution even during ‘sterile’ inflammation [40]. IL-18 can facilitate infiltration of multiple circulating immune cells through the BBB including monocytes [19, 22, 24], neutrophils [22, 23], and T-cells [25]. In addition, infiltration is augmented through cell adhesion molecule expression on endothelial cells [15, 22, 26]. This study shows that neutrophil infiltration occurs in injured brain regions following seizurogenic nerve agent exposure. However, increases in neutrophil infiltration and CXCL1 expression precede significant expression of IL-18, which does not correlate with neutrophil infiltration (except in the thalamus) or with CXCL1 expression. In contrast, previous studies have shown that CXCL1 positively correlates to neutrophil infiltration with high significance [9]. In addition, T-cells respond to the primary excitotoxic injury within the first 24 h, though this is also prior to significant IL-18 expression. While IL-18 may be involved in T-cell activation, the rapid reduction in T-cells in the injured tissue by 48 h and the distinct lack of all primary T-cell expressed factors such as IL-4, IL-10, IL-12, IL-17 and IFNγ [8] suggest that these cells do not become activated or participate substantially in the progressive lesion formation seen in this model at the time points investigated. It is unlikely that IL-18 contributes to these acute events in this model since both neutrophils and T-cells infiltrate into the tissue prior to significant IL-18 expression. However, IL-18 can induce neutrophils to produce a myriad of inflammatory factors and may play a role in activation after 72 h once the cells have infiltrated the injured tissues [23].

## Conclusions

This study showed a significant increase in IL-18 in brain regions injured by excitotoxic seizure activity, starting as early as 24 h after GD exposure. IL-18 expression largely originated from endothelial cells particularly those around areas of vascular damage. The expression significantly correlates with the appearance of activated macrophages in the brain but not to T cells or, from previous data, neutrophils. This study did not find evidence that IL-18 is integrally involved in a propagation of the adaptive immune response in the time frame investigated and may therefore be primarily contributing to amplification of the acute innate inflammatory response. In addition, this expression may be part of the angiogenic mechanism to repair damaged vasculature caused by micro-hemorrhage formation in the brain in response to GD exposure though this mechanism does not appear to be active concurrent with IL-18 expression. Expression of IL-18 within the first 48 h may therefore be related to leukocyte infiltration and/or delayed angiogenesis, though further studies are needed to elucidate this role.
